# Apocynum Cannabium in Bright’s Disease

**Published:** 1881-06

**Authors:** T. S. Dabney

**Affiliations:** No. 388 Magazine Street; New Orleans, La.


ARTICLE II.

### APOCYNUM CANNABINUM IN BRIGHT'S DISEASE.

BY T. S. DABNEY, M. D., NEW ORLEANS, LA.

Editor INDEPENDENT PRACTITIONER:

In December, 1880, I read a paper before the New Orleans Medical and Surgical Association, calling attention to the therapeutic value of the Apocynum Cannabinum. My paper was ordered printed by the association and appeared in the February, 1881, number of the N. O. Med. and Surg. Journal. Believing the Apocynum to be the most reliable and least dangerous of all hydrogogue cathartics and diaphoretics, and knowing that your columns are always open for anything that tends to alleviate human suffering and prolong life, I beg you to give to your numerous subscribers the results of my three years experience with this now almost forgotten remedy.

As visiting physician to the Charity Hospital of New Orleans, I have had every opportunity for testing the remedy in every stage of nephritis. I will state that I have never tested personally any of the apocynum kept in drug stores, knowing that, like Cannabis Indica—one of the same family—it rapidly deteriorates with age. All of my experiments were made with a tincture made from fresh roots sent me from Mississippi, in the proportion of one ounce of the root to six ounces of dilute alcohol. Dose from five to thirty drops.

As this remedy has not received its proper amount of attention from writers on therapeutics, you will excuse me for stating briefly its properties. In small doses it is tonic. In larger doses, it is antiperiodic, diuretic, diaphoretic and cathartic. In large doses emetic. Profuse catharsis can usually be obtained from twenty drop doses, thrice repeated on an empty stomach during the day. Thirty drops is usually followed by free emesis. Its cathartic action is marked by but little tormina or tenesmus. Its action on the heart is similar to that of digitalis, and my experiments with it in acute degenrative nephritis would lead me to infer that its diuretic properties are of an extrarenal character, that is to say, it acts as a diuretic by blood pressure. In this article I will not pretend to deal with nephritis from its pathological and symtomatological aspect, but will content myself with its therapeia. In order to show thoroughly the therapeutical value of apocynum cannabinum, I will report several cases, which are taken from my original article. I prefer to take hospital cases, inasmuch as they are less liable to error that private ones; besides, hospital cases are of public record.

CASE I. George B., aet. 20, native of Mississippi, was admitted to ward 25, October 23, 1879. Upon admission paitient presented that peculiar whitish appearance almost pathognomonic of chronic nephritis. Upon examination the urine was found to be heavily charged with albumen, and casts in abundance were found under the microscope. In November I assumed charge of the case, through the courtesy of Prof. Jno. B. Elliott, though I saw no hopes of any ultimate good. The patient was suffering from general anasarca, which Prof. Elliott had failed to remove by means of the usual diuretic and cathartic remedies. The patient was at once put on Tr. Apocyn. Cannabin., gtt. xx ter die, which was increased gradually to gtt. xxx. The urine soon became abundant, a quart or more being passed daily. The patient had from fifteen to twenty watery operations daily. In a few days the œdema of face, arms, legs, and feet disappeared. Examination of the urine showed no diminution in quantity of albumen or casts. The dropsy never returned, though the patient's stomach refused the drug after an emetic dose of it had been taken.

On December 26th, 1879, the patient at his request was discharged. He returned home and died shortly afterwards.

CASE II. Wm. W., aet. 29, native of Alabama, was admitted to Ward 22, January 23, 1880. Patient had lived many years in the Louisiana swamps, and his constitution was shattered by malarial fevers. His spleen extended beyond the median line. On admission patient presented general anasarca to an enormous extent; great puffiness under the eyes; pale and anæmic in appearance. Patient passed about four or five ounces of urine daily. Pulse weak, but hard, showing thickening of arterial coats. Patient attributed his disease to a severe cold which was attended by severe pain in the loins and dropsy. Urine abounded in albumen and casts. Patient complained of excruciating cephalalgia, due doubtless to uræmia. Considerable lumbar pain present also. Diagnosis: chronic nephritis. Jan. 24, patient was put on Trac Apocyn. Cann., gtt. xx ter die, and

Liq. Pot. Arsenit. gtt. v., Cinchonidia gr. v, ter die. Jan. 25, no change except three watery passages during the night. Jan. 26, Urine very copious; catharsis moderate; patient feels much better. The ascites, which was enormous, is much reduced. Jan. 27. Marked diminution of dropsy; diaphoresis and diuresis most copious. Bowels acted twice in twenty-four hours. Pain lessened in loins, remains in head. Jan. 28. Several quarts of urine were voided in the last twenty-four hours; two passages. Reduction of dropsy continues; no pain in loins, but little in the head. Appetite is voracious. The patient is steadily gaining strength. Jan. 29. One passage; urine copious. To-day the arsenic and cinchonidia were discontinued. On the 28th the Apocynum vomited the patient; since then the dose has been reduced to ten drops. Feb. 2. The patient not having had a trace of dropsy for many days, his urine being nearly destitute of albumen and casts, his appetite being excellent and his general health being good, he was this day, at his earnest request, discharged, much against my will; for I well knew that the disease was only checked, not cured.

On May 11, the patient returned in the same condition as he was upon his first admission. Twenty drops of Apocyn. Cann. thrice daily were administered. From this date to the 17th patient had twenty to thirty passages a day. On this date no dropsy was present, but little albumen, but an abundance of casts. June 24. Patient left agaiast my wish, being strong and vigorous looking. July 24. Patient returned in same condition as before. The Apocynum removed all dropsy in a few days, and caused the skin and kidneys to act well.

Oct. 22. No dropsy for two months; no casts and only traces of albumen can be found about once a week. Patient takes five drop doses of the medicine daily. Patient left the hospital about the middle of November apparently cured. He has not returned up to date.

Case III. Acute Desquamative Nephritis. Charles T., aet. 35, was admitted to Ward 26, April 9th, 1880. Puffy under eyes; great ascites; œdema of feet and legs; urine abounding in albumen, epithelial casts and oil globules. Ordered turpentine stupe and half ounce of sulphate of magnesia. Tr digitalis, in ten-drop doses, thrice daily, was given for two days, after which time nothing but the apocynum was given. In ten days all traces of dropsy, albumen, casts and oil globules had disappeared. April 22d patient was discharged strong and vigorous.

Case IV. Sub-acute Nephritis. Patrick C., nurse in Ward 24 for a long time. He is still the nurse there. Patrick is sixty years old.

For six months the insidious disease had been slowly undermining Patrick's health. An able physician had in vain striven to relieve Patrick of his dropsy and its attendant evils. On April 16, 1880, I assumed charge of the case. Patrick's condition was as follows: Enormous general anasarca of some months' standing. About three ounces of urine, laden with casts and albumen, was passed daily. Patient suffered greatly from dyspnœa and anorexia.

On May 4th no casts, albumen or dropsy could be detected. Patient's appetite became good. The apocynum was continued in five-drop doses for one month longer. Frequent examinations of the urine were made, but neither casts nor albumen were ever found in it afterwards. Over one year has elapsed now, and the patient is hale and hearty, and at his post as nurse.

The above four cases, taken at random from a large number, will serve to show the value of apocynum in all the various grades of nephritis.

Dr. T. T. Meade, an eminent physician of Mississippi, has been using, with uniform success, for the past twenty-five years, the apocynum for the relief of all forms of dropsy. Dr. Meade also states that it is an anti-periodic, scarcely, if any, inferior to the cinchona salts.

Dr. Wm. E. Brickell, one of the most prominent physicians in this city, used it, with most satisfactory results, in seven cases of anasarca due to scarlet fever. It is unnecessary to mention the names of others that have lately given the apocynum a trial. I feel sure that no physician who has ever used it once could fail to perceive the wonderful rapidity with which dropsical effusions disappear under its use. Its fine tonic properties compensate, in a measure, for its hydragogue properties. Patients invariably commence gaining strength during its administration, even when they are being severely purged.

The medicine, when given in small doses, and after repeated, produces tennitus aurium to a painful degree. It always produces a sensation of tingling and warmth in the extremities. Its utter harmlessness, coupled with its potency, must ever commend it as the safest and most reliable of all hydragogues.

No. 388 Magazine Street, May 18, 1881.

